# Effects of tight *versus* non tight control of metabolic acidosis on early renal function after kidney transplantation

**DOI:** 10.1186/2008-2231-20-36

**Published:** 2012-09-10

**Authors:** Farhad Etezadi, Pejman Pourfakhr, Mojtaba Mojtahedzade, Atabak Najafi, Reza Shariat Moharari, Kourosh Karimi Yarandi, Mohammad Reza Khajavi

**Affiliations:** 1 Sina Hospital, Tehran University of Medical Sciences, Hassan Abad sq, Tehran, Iran

**Keywords:** Acid–base disorder, Renal transplantation, Chronic renal failure, Sodium bicarbonate

## Abstract

**Background:**

Recently, several studies have been conducted to determine the optimal strategy for intra-operative fluid replacement therapy in renal transplantation surgery. Since infusion of sodium bicarbonate as a buffer seems to be safer than other buffer compounds (lactate, gluconate, acetate)that indirectly convert into it within the liver, We hypothesized tight control of metabolic acidosis by infusion of sodium bicarbonate may improve early post-operative renal function in renal transplant recipients.

**Methods:**

120 patients were randomly divided into two equal groups. In group A, bicarbonate was infused intra-operatively according to Base Excess (BE) measurements to achieve the normal values of BE (−5 to +5 mEq/L). In group B, infusion of bicarbonate was allowed only in case of severe metabolic acidosis (BE ≤ −15 mEq/L or bicarbonate ≤ 10 mEq/L or PH ≤ 7.15). Minute ventilation was adjusted to keep PaCO_2_ within the normal range. Primary end-point was sampling of serum creatinine level in first, second, third and seventh post-operative days for statistical comparison between groups. Secondary objectives were comparison of cumulative urine volumes in the first 24 h of post-operative period and serum BUN levels which were obtained in first, second, third and seventh post-operative days.

**Results:**

In group A, all of consecutive serum creatinine levels were significantly lower in comparison with group B. With regard to secondary outcomes, no significant difference between groups was observed.

**Conclusion:**

Intra-operative tight control of metabolic acidosis by infusion of Sodium Bicarbonate in renal transplant recipients may improve early post-operative renal function.

## Background

Kidney transplantation is a cardinal method and the most cost-effective treatment modality used for the patients with chronic renal failure (CRF) [1].

Experiments in animals show that premedication with Sodium Bicarbonate, before the development of ischemic damage in renal tubules, may have renoprotective effects [2,3]. It is obvious that transplanted kidney is under the risk of ischemic insult (warm and cold ischemic period).Furthermore, Infusion of sodium bicarbonate solution (8.4%), which is hyperosmolar, can shift intracellular water into interstitial and intravascular spaces leading to intravascular volume expansion and induces osmotic diuresis [4-6]. Meanwhile, the alkalization of the urine might increase the solubility of acidic materials accumulated in CRF patients and enhance their excretion through urine [7]. The main concern with bicarbonate infusion is CO2 retention in the body. In other words, every 100 mEq of bicarbonate can produce 2.24 l of CO_2_ in the body. This amount equals to an average adult CO2 production during 10 min of normal activity [8]. The extra load of CO_2_ can be exhaled by increasing pulmonary minute ventilation which could be easily achieved during general anesthesia.

During recent years, several studies have been conducted to determine the optimal strategy for intra-operative fluid replacement therapy in renal transplantation surgery [9-11]. Three types of crystalloids have been examined for this purpose.1- Normal saline which is the preferred IV fluids for administration during kidney transplant surgery [12]. Infusion of large-volume of NS during renal transplantation can lead to hyperchloremic acidosis. 2-Lactated Ringer’s solution which contains lactate anion, and can be transformed into bicarbonate in the liver; its infusion prevents aggravation of metabolic acidosis and so, produces less hyperkalemia in comparison with normal saline solution. 3- Plasmalyte solution contains acetate and gluconate which can be converted to bicarbonate in the liver. It is obvious that a normal functional liver with adequate hepatic blood flow is a prerequisite for this buffer effect attributed to the aforementioned balanced salt solutions. On the other hand, it has been already shown that anesthetic agents might reduce the hepatic blood flow [13].

Additionally, lactate may be pro-inflammatory by itself [14]. Also, lactated Ringer’s solution has been reported to induce hypercoagulable state [10,15].

With regard to the above-mentioned facts and relatively safe profile of Sodium bicarbonate, and lack of a clinical trial in literature which deals with acid–base balance during a renal transplant surgery we designed this study based on the following hypothesis: The adjustment of metabolic acidosis with infusion of Sodium bicarbonate, even before the implantation of the kidney may reduce the work-load of ischemic donor’s kidney and as a result, may improve the early outcome of the transplanted kidney.

## Methods

After receiving the approval of the ethics committee of Research Deputy of our University, a written informed consent was obtained from all eligible patients who were candidate for living-donor renal transplantation. After study registration at IRCT website as: "IRCT138902163829N2",this prospective study was conducted on 120 patients with American Society of Anesthesiologists (ASA) class 3 and 4 who underwent renal transplantation from August 2010 to August 2011 in urology and transplantation operating room of Sina Hospital in which about 8-12 kidney transplantations are being done monthly. The randomization was performed by using a computer generated table of random numbers. The inclusion criteria are patients of any age who are candidate for elective kidney transplantation. The exclusion criteria were severe congestive heart failure (EF ≤ 35%), recent use of acetazolamide (during the past 24 h), detection of serum potassium level higher than 6 mEq/L and lower than 2.9 mEq/L, and serum sodium level higher than 155 mEq/L. Occurrence of severe hypotension (SBP ≤ 90 mmHg) indicates either more rapid fluid replacement than the protocol of the study or need for catecholamine infusion due to uncontrolled surgical bleeding. Therefore, it was another exclusion criterion of this study.

Patients, anesthesiologists and other anesthesia team members, who were responsible for postoperative evaluation of primary and secondary outcomes, were blinded to intra-operative interventions. All recipients had been undergoing hemodialysis the day before surgery. Standard monitoring according to the recommendations of ASA was used. For all patients radial arterial cannula was inserted before the induction of anesthesia in order to monitor blood pressure and obtain blood samples. Central venous catheter was inserted in the right internal jugular vein after induction of anesthesia for infusion of crystalloids and Sodium Bicarbonate and central venous pressure (CVP) monitoring in the patients.

General anesthesia was induced with a combination of IV midazolam (0.05 mg/kg), fentanyl (2 μg /kg) and sodium thiopental (4 mg/kg). Anesthesia was maintained using isoflurane in an air/oxygen mixture and the bolus injection of fentanyl (2 μg/kg) every hour; muscle relaxation was achieved by use of IV injection of atracurium (0.2 mg/kg every 20 min).

Intra-operative fluid replacement therapy was performed according to the following protocol: Every patient received 20-25 ml/kg/h of only NS titrated continuously during the anesthesia, while CVP was kept between10-15 cm H2O. All eligible patients were randomized into two groups, each of them consisting of 60 cases; the intervention group of patients (group A) was scheduled to receive sodium bicarbonate infusion (8.4%) for tight control of metabolic acidosis according to Base Excess (BE) measurements, which were determined by an arterial blood gas analyzer (Gem primer 3000, Instrumentation laboratory, USA) from the commencement of anesthesia. The first arterial blood sample was obtained *via* arterial cannula just after induction of anesthesia and then, every 30 min up to the end of operation.

In the case of BE lower than−5 mEq/L, the clinician was permitted to start sodium bicarbonate infusion to keep the BE values in normal range (between +5 mEq/L to−5 mEq/L) [8,16,17]. Accordingly, if the BE value was below−5 mEq/L, the total deficit of bicarbonate was calculated with respect to the volume of distribution of sodium bicarbonate (30% of body weight), and half of the total deficit was infused through the central venous catheter during 15 min in the group A [8].

In the control group (group B), the infusion of sodium bicarbonate was allowed only in case of severe metabolic acidosis (BE ≤−15 mEq/L or serum bicarbonate level ≤ 10 mEq/L or PH ≤ 7.15).

Blood products were administered, when clinically indicated based on ASA recommendations. Every unpredicted complication occurring during anesthesia was treated by the clinician caring for the patients.

The patients were ventilated on continuous mandatory ventilation (CMV) mode according to this initial setting: RR = 10, TV = 10 cc /kg, I/E ratio = 1/2.

The ventilator setting (RR or TV) was adjusted every 30 min (after observing ABG result) by the clinician responsible for patients’ care to keep the PaCO_2_ between 35 to 40 mmHg. Perioperative immunosuppressive therapies were administered to all patients according to our institutional protocol:

1. Mycophenolate mofetil (Cell Cept): 1-2 g

2. Cyclosporine (Neoral): 6.5-7 mg/kg

3. Prednisolone: 2 mg/kg

All of the transplantation surgeries were performed by the same surgical team including an urologist and a vascular surgeon. All of the donors received 0.25gr/kg of mannitol infusion just before procurement of the left kidney *via* an open approach. The donor kidneys were flushed with lactated Ringer’s solution before being transferred to the operating room. Afterwards, the kidneys were implanted in the right or left retroperitoneal space of the recipients. All the recipients received 5,000 units of heparin intravenously (three minutes before performing the clamp). All patients received 5 mg/kg of furosemide just after de-clamping of the implanted kidney as a routine intervention. The mean arterial pressure (MAP) of the patients was recorded just before and 15 min after the injection of furosemide. Postoperative IV fluid therapy was the same for all of the patients as the following protocol: Urine output was replaced (one milliliter for one milliliter) with an IV infusion of dextrose 5%/0.45% Na Cl plus 20 mEq/L of Sodium Bicarbonate.

Our primary endpoint was to evaluate the effects of metabolic acidosis tight control while maintaining the normal intraoperative PaCO_2_ on the postoperative early renal function through four consecutive sampling of the serum creatinine levels, which were measured on first, second, third and seventh postoperative days. As the secondary objective, the effect of intra-operative bicarbonate infusion on the urine volume and serum BUN level in the early postoperative period was evaluated. Serum BUN concentration levels were measured in the first, second, third, and seventh days of the postoperative period. Cumulative urine volumes in the recovery room and during the first six and twenty four hours after the surgery were recorded as well.

### Statistical analysis

Data are presented as means ± SD for continuous variables and as percentages for categorical variables. A sample size of 60 in each group was calculated to have at least 80% power to detect the expected difference between treatment protocols with respect to the primary goal. A 0.7 mg/dl difference in the mean serum creatinine level was noted to be significant. All data were tested for normality using method of Kolmogorov-Smirnov. Sphericity assumption was checked by Mauchly test before comparisons. Different variants of multiple measurements were separately analyzed using GLM repeated measurement analysis. A p value less than 5% was considered significant. All the laboratory measurements were analyzed at the central laboratory of Sina University Hospital.

## Results

**Figure 1:**
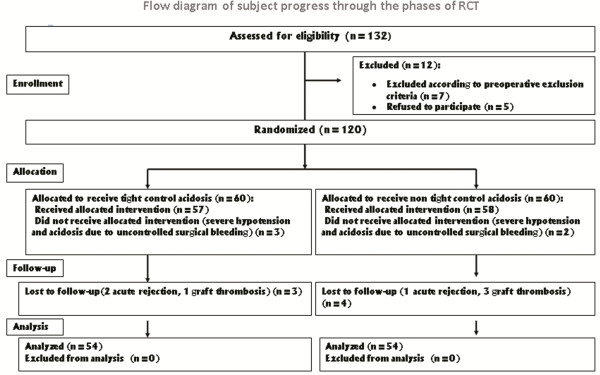
Flow diagram of subject progress through the phase of RCT.

One hundred twenty patients were randomized into two equal groups. Demographic characteristics and preoperative variables were comparable in the two groups (Table 1). In the intra-operative period, three patients in group A were excluded from the study because of severe surgical bleeding and life threatening hypotension. This event occurred for two of the patients in the group B. The follow up could not be indicated in three other patients of group A because of two biopsy proven acute rejections and one Doppler sonography proven graft thrombosis. Meanwhile, three cases of graft thrombosis and one case of acute rejection were also observed in group B. At last, data from fifty four patients in each group were gathered and underwent statistical analysis. Flowchart of the study progress is depicted in Figure 1.

**Table 1:** Comparison of demographic and preoperative variables of both groups

**Variable**		**Group A**	**Group B**
		**Mean ± SD**	**Mean ± SD**
Sex	Male	36 (66%)	33 (61%)
	Female	18 (34%)	21 (39%)
Age (yrs.)		42 ± 15	44 ± 15
Weight (kg)		65.6 ± 13.2	66.3 ± 20.6
Dialysis before surgery (h)		23 ± 4	23 ±3
Baseline Hgb (g/dl)		11.9 ± 3.1	10.1 ± 1.9
Baseline Albumin (mg/dl)		3.9 ± 0.3	4.1 ± 0.2
Baseline MAP in operating room (mmHg)		110 ± 14	114 ± 13
Baseline PH		7.35 ± 0.04	7.34 ± 0.07
Baseline BE (mEq/L)		−4.4 ± 0.2	−4.6 ± 0.3
Baseline PaCO2 (mmHg)		35 ± 1.5	34 ± 1.6
Baseline Cr (mg/dl)		5.1 ± 1.3	5.1 ± 1.2
Baseline BUN (mg/dl)		90.0 ± 32.4	92.1 ± 33.1

[Data are reported as mean ± SD] Hgb = hemoglobin, BP = blood pressure, mEq/L = milli equivalent per litre, MAP = mean arterial pressure, BE = base excess, Cr = creatinine, Pa CO2 = arterial pressure of CO2 gas, BUN = blood urea nitrogen.

All patients received similar volumes of NS as fluid replacement therapy during the surgery (5.54 ± O.51 *vs.*5.70 ± 0.67 lit) and no patient received colloid or blood products throughout the transplantation. No significant difference exists regarding to the duration of surgery between the groups. Mean amount of bicarbonate infused during the surgery was 87.4 ± 50.7 mEqin group A. Since, mean change of BE during the surgery was −6 ± 2.7 mEq/Lin group B, there was no indication for sodium bicarbonate infusion in this group. Duration of warm and cold ischemia and the mean amount of bleeding were similar among the groups. Mean of PH values at the end of surgery was 7.39 ± 0.05in group A and it was 7.20 ± 0.03in the group B while, the mean of multiple PH measurements throughout the operation was 7.38 ± 0.04 in group A and it was 7.21 ± 0.05 in group B. In addition, mean of BE at the end of surgery in the group A was −1.6 ± 1.3 and it was −10.6 ± 2.9in the group B. MAP of the patients decreased 15 min after furosemide injection in both groups, but this decline was temporary and self-limited (Table 2).

**Table 2:** Comparison of intra-operative variables between groups

**Variable**	**Group A**	**Group B**
	**Mean ± SD**	**Mean ± SD**
Time of surgery (min)	222 ± 30	227 ± 31
Amount of sodium bicarbonate infused (mEq)	87 ± 50	0
Extent of BE change during operation (mEq/L)	_	−6 ± 2.7
Amount of NS infused during surgery (lit)	5.5 ± 0.5	5.7 ± 0.6
Time of cold ischemia (min)	3.9 ± 2	4.01 ± 2
Time of warm ischemia (min)	18.3 ± 1.9	18.1 ± 2.1
Blood loss (ml)	294.2 ± 112.8	291.1 ± 103.9
MAP of patients before furosemide injection	138 ± 24	119 ± 32
MAP of patients 15 min after furosemide injection	122 ± 15	109 ± 18
Mean of multiple PH values throughout operation	7.38 ± 0.04	7.21 ± 0.05
Mean of PH value at the end of surgery	7.39 ± 0.05	7.20 ± 0.03
BE at the end of surgery(mEq/L)	−1.6 ± 1.3	−10.6 ± 2.9

[Data are reported as mean ± SD] BE = base excess, NS = normal saline, MAP = mean arterial pressure, mEq/L = milli equivalent per litre.

We observed significantly lower serum creatinine level in the group A in comparison with the group B throughout the days of follow up (p = 0.001) (Table 3).

**Table 3:** Comparison of primary and secondary outcomes between groups

**Measurements**	**Group A**	**Group B**	**P Value**
	**Mean ± SD**	**Mean ± SD**	
Urine volume in Recovery (ml)	812 ± 492	377 ± 384	P = 0.075
Total Urine volume in 6 h (ml)	3696 ± 2401	1578 ± 1422	
Total Urine volume in 24 h (ml)	11279 ± 3937	9960 ± 5718	
Cr (mg/dl) 1^st^ day	3.2 ± 0.8	3.5 ± 1.3	P = 0.001
Cr (mg/dl) 2^nd^ day	1.7 ± 0.6	2.7 ± 1.2	
Cr (mg/dl) 3^rd^ day	1.5 ± 0.5	2.5 ± 0.9	
Cr (mg/dl) 7^th^ day	1.4 ± 0.3	2.2 ± 0.8	
BUN(mg/dl) 1^st^ day	67.6 ± 21	79.2 ± 40	P = 0.74
BUN(mg/dl) 2^nd^ day	56.4 ± 24.8	76.2 ± 48.7	
BUN(mg/dl) 3^rd^ day	55.51 ± 30.3	81.11 ± 51.9	

[Data are reported as mean ± SD] Cr = creatinine, BUN = blood urea nitrogen.

The cumulative urine volumes gathered in the first 24 h of our study in both groups were not significantly different (p = 0.075) (Table 3).

Serum BUN levels were measured four times throughout the study and in all of them, lower BUN levels were observed in the group A, but the difference was not statistically significant(p = 0.74) (Table 3).

## Discussion

According to meticulous literature search, this study is the first one which evaluates effects of intra-operative metabolic acidosis tight control on early renal function after a kidney transplantation surgery.

The development of hyperchloremic metabolic acidosis during intra-operative period is a well-recognized complication attributed to infusion of large volumes of NS solution [18,19]. It is worthwhile to mention that other types of anions (sulfate, phosphate, formate, etc.) have usually accumulated in CRF patients, but crystalloid replacement therapy doesn’t change their plasma levels except some dilution that may ensue, and also they have a minor role in acid–base balance in comparison to such an abundant and strong anion like chloride. On the other hand, infusion of large volumes of crystalloids is a crucial strategy during a renal transplantation surgery [20]. Therefore, occurrence of hyperchloremic acidosis is unavoidable in these cases. With respect to normal range of serum chloride level (102-106 mEq/L) and the mean amount of NS solution infused (Table 2), every patient in this study received approximately 250 mEq extra load of chloride, which distributes throughout the extracellular fluid compartment [8]. Accordingly, after final equilibrium, serum chloride level is anticipated to raise about 10 mEq/L .According to Stewart-Fencl approach, 10 mEq/L of chloride excess results in 10 mEq/L decrease in Strong Ion Difference (SID) [16,17]. Consequently, such a decline in SID, reduces 10 mEq/L of the BE value measured by the ABG analyzer [16,17,21]. On the other hand, infusion of about 5l of NS during the surgery leads to 25% dilution of serum albumin concentration which is the main part of serum “A total”(an independent variable which includes sum of negative charges of weak anions such as phosphate and albumin). Hence, about +3 mEq/L increment in BE value takes place [16,17].

Thus, an acidifying force of about-10 mEq/L (hyperchloremia) faces an alkalinizing force of about +3 mEq/L (hypoalbuminemia).The final effect would be about-7 mEq/L of acidifying force. It must be mentioned that there are a few papers in the current literature which deal with the issue of optimal crystalloid therapy in a renal transplant surgery [9-11,22-26].

The effects of large-volume infusion of NS versus lactated Ringer’s solution were compared by O’Malley et al. in renal transplant patients [9].They stated that lactated Ringer’s solution may be as safe as NS. They incidentally found (through an intra-group analysis in NS group) a significantly higher urine volume and lower serum creatinine level in eight patients who were treated with bicarbonate to adjust metabolic acidosis. Accordingly, they suggested that metabolic acidosis was the probable reason of such a negative impact on renal functions in those patients who were not treated for acidosis. As they stated in their article, they did not define an algorithm for treatment of metabolic acidosis. So, to avoid the mentioned shortcoming, we designed a definite algorithm for treatment of metabolic acidosis in those who underwent a large volume infusion of NS. Surprisingly, our results support their findings regarding to beneficial effects of the metabolic acidosis control on a renal function. Several investigators have already suggested that hyperchloremia by itself may be the cause of such a detrimental effect on a renal function [22-24]. It must be pointed out that we normalized metabolic acidosis without enhancing excretion of extra load of chloride ion. Despite it, better early renal function tests were achieved. This finding may reveal that acidosis on its own, and not the chloride ion excess by itself is the cause of harmful effects on a renal function.

Hadimioglu et al. observed significantly larger urine output in NS group in comparison with lactated Ringer’s and Plasmalyte solution groups in spite of significantly progressive rise of serum chloride level observed in the NS group [11]. However, they didn’t find any significant difference between groups in terms of the renal function tests after the surgery. Their findings may support our concept that hyperchloremia on its own may be an innocent factor. Recently, Modi MP et al. through a letter to the editor stated that lactated Ringer’s solution is as safe as NS in a renal transplant surgery [25]. Othman et al. have a different view point; they investigated on rapidity of fluid replacement therapy instead of the type of fluid which is used. They suggested that maintaining a given CVP in the renal transplant patients as a target by adjusting NS infusion rate, is superior to constant continuous infusion of NS in terms of an early post-operative renal function [26]. Another point that must be clarified is a probable negative impact of vasodilation and resulting hypotension induced by Lasix on the renal perfusion. Our results show a little reduction in MAP of both groups which seems clinically unimportant. The reason may be that vessels have been already dilated by anesthetic drugs in patients under general anesthesia, so minimal vasodilation is anticipated in these patients. In addition, equal amount of Lasix is injected to both groups, thus, it could not be regarded as a confounding factor in this study.

Our study may be subject to a number of limitations. 1-Only living donor transplantations were enrolled in this study. Thus, our results are merely applicable to this group of patients. 2- The other criticism is that a traditional marker (consecutive serum creatinine levels) was used as an indicator of an early renal function [27]. Because of logistical reasons, we could not use novel biomarkers of kidney injury like β2 Microglobulin, α- GluthationeS-transferase, etc. for this purpose. Although, the superiority of novel biomarkers over traditional ones is still unproven, use of novel biomarkers especially those which are specific for evaluation of ischemic renal insult may be advantageous in the transplanted kidney that is under such a risk.

## Conclusion

Intraoperative metabolic acidosis tight control by using Sodium bicarbonate infusion, while keeping PaCO_2_ level in normal range, may be an advantageous strategy to enhance the early postoperative kidney function in a renal transplant surgery.

## Competing interest

The authors declare that they have no competing interests.

## Authors’ contributions

FE: Reviewing the literature, designing the study method, preparing the manuscript and final revision. PP: Conducting the study and gathering the data, preparing data for analysis. MM: Contributing to design the study method and interpreting the study results. AN: Participating in literature review and designing the study method. RSM: Conducting the study, participating in the analysis of data. KKI: Contributing to write the manuscript. MRK: Designing the study method and the questionnaire, conducting the study, interpreting the results. All authors read and approved the final manuscript.
